# A Regulatory Network of Arabinogalactan Proteins, Glycosylation, and Nucleotide Sugars for Optimizing Mara des Bois Strawberries Postharvest Storage Quality

**DOI:** 10.3390/plants14172796

**Published:** 2025-09-06

**Authors:** María Isabel Escribano, Irene Romero, María Teresa Sanchez-Ballesta, Carmen Merodio

**Affiliations:** Department of Characterization, Quality and Safety, Institute of Food Science, Technology and Nutrition (ICTAN-CSIC), Jose Antonio Novais 6, 28040 Madrid, Spain; irene.romero@ictan.csic.es (I.R.); mballesta@ictan.csic.es (M.T.S.-B.)

**Keywords:** CO_2_ pretreatment, *Fragaria* sp., galactosyltransferases, immunocytolocalization, *O*-glycoproteins, prolyl 4-hydroxylase, postharvest storage, transcriptional and post-translational regulation, senescence, UDP-sugars

## Abstract

Arabinogalactan proteins (AGPs) and extensins influence cell wall assembly and regulate plant cell mechanical properties through interactions with extracellular matrix polymers. These proteins may play a key role in the biochemical events underlying postharvest treatments aimed at controlling fruit texture and turgor loss associated with senescence-related disorders. We studied the temporal and spatial accumulation patterns of extensin and AGP isoforms constitutively expressed along with the profiling of nucleotide sugars UDP-galactose, UDP-arabinose, UDP-glucuronic acid, and UDP-rhamnose in Mara des Bois strawberries under different storage conditions. We also assessed the expression timing of AGP-encoding genes (*FvAFP4*, *FvAGP5*) and genes involved in key steps of post-translational glycosylation (*FvP4H1*, *FvGAT20*, *FvGAT7*). Whereas extensins are down-regulated, AGPs are transcriptionally regulated by cold and cold-high CO_2_ and post-translationally modulated after transfer to 20 °C. Based on their subcellular localization, molecular properties, isoform-specific glycosylation, UDP-sugar availability, and timing-regulated expression, AGPs are likely involved in cell wall assembly and modulation of mechanical properties. Consequently, they may influence fruit texture and enhanced softening resistance, potentially counteracting senescence-associated disorders through CO_2_-responsive signaling mechanisms. Conversely, the decrease in both UDP-galactose levels and *AGPs* gene expression in non-cold-stored senescent strawberries at 20 °C further supports their relevance in AGPs biosynthesis regulation and underscores their potential as markers for improving postharvest storage strategies.

## 1. Introduction

Arabinogalactan proteins and extensins, along with lectins and proline-rich proteins, are *O*-glycosylated proteins that belong to the extracellular class of intrinsically disordered proteins within the hydroxyproline (Hyp)-rich *O*-glycoproteins (HRGPs) superfamily. These proteins are particularly rich in proline (Pro), an amino acid that promotes disorder due to its rigid conformation. They also contain motifs that direct post-translational modifications [1].

Arabinogalactan proteins (AGPs), the most highly glycosylated members of the HRGPs family, are complex cell surface *O*-proteoglycans. They contain type II AG glycan moieties attached to non-contiguous hydroxyproline (Hyp) residues, with a β-(1 → 3)-galactan backbone substituted at C(*O*)6 with side chains of β-(1 → 6)-galactan of variable length. These side chains are further decorated with arabinose, and, less frequently, with rhamnose and glucuronic acid. The biosynthesis and biological function of AGPs are tightly regulated at transcriptional and post-translational levels. Once amino acids are assembled into peptide chains, an N-terminal signal peptide directs AGPs to the endoplasmic reticulum, where proline hydroxylation occurs. A glycosylphosphatidylinositol (GPI) anchor may also be added to the hydrophobic C-terminal domain. Proline hydroxylation is a prerequisite for the *O*-glycosylation of cell wall proteins at Hyp residues. This process begins in the endoplasmic reticulum, where selected proline residues are converted into Hyp residues by a multigene family of enzymes known as prolyl 4-hydroxylases (P4Hs), which provide the reactive hydroxyl groups necessary for *O*-glycosylation [2,3,4].

Both GPI-anchored and unanchored AGPs are then transferred to the Golgi apparatus, where extensive glycosylation occurs through the action of specific glycosyltransferase enzymes. These enzymes play a central role in determining the AGP glycosylation profile, which influences their functionality. The biosynthesis process is initiated by hydroxyproline galactosyltransferase, which attaches the first UDP-galactose residue to Hyp in the protein core. The activity of β-1,3-galactosyltransferase and β-1,6-galactosyltransferase then added UDP-galactose to the β-1,3-galactan backbone and the β-1,6-galactan side chains of AGP glycans. Arabinosyltransferase catalyzes the attachment of UDP-arabinose residues to the β-1,3-galactan backbone, while glucuronosyltransferases add UDP-glucuronic acid to both the main backbone and side chains of AGPs [5,6].

After glycosylation, AGPs are secreted to the cell surface via vesicles, where they are either deposited in the periplasm or cell wall or released into the apoplast. This complex and diverse class of glycosylated proteins plays a key role in cell wall assembly by cross-linking with other cell wall glycopolymers and forming calcium bridges between molecules. AGPs significantly influence cell wall mechanics and signaling by interacting with extracellular matrix polymers and plasma membrane proteins, thereby regulating dynamic developmental processes and responses to environmental changes [7].

Extensins are highly arabinosylated *O*-glycoproteins characterized by continuous Hyp residues found in Ser-Hyp_3-5_ repeats, also known as extensin motifs. These residues are modified with *O*-arabinofuranose, and oligoarabinose structures are formed through the addition of multiple arabinose residues. Following β-arabinofuranosylation of the Hyp residues, oligoarabinose structures are formed [8]. Similarly to AGPs, extensins undergo two key post-translational modifications following synthesis of the protein backbone: the hydroxylation of specific proline residues to 4-hydroxyproline by prolyl 4-hydroxylases (P4Hs) and the subsequent *O*-glycosylation of the adjacent 4-hydroxyproline residues by glycosyltransferases involved in adding arabinoses to short arabinoside chains and a single galactose to serine residues. Following *O*-glycosylation, extensins are secreted into the cell wall, where they form an ordered network linked by covalent bonds between tyrosine residues, mediated by specific peroxidases. This cross-linked extensin network is thought to be essential for cell wall assembly and growth, facilitating cell extension and expansion. It also strengthens the glyco-network, enhancing the physical properties of the cell wall and providing mechanical protection against pathogens. Therefore, it plays an essential role in establishing and maintaining cell wall structure [9,10].

The goal of proper postharvest handling is to preserve optimal fruit quality for as long as possible after harvest by slowing down the rate of senescence and the development of rots, particularly during the transfer to ambient temperature at the time of consumption. This is relevant for strawberries, a non-climacteric fruit that is particularly vulnerable to the rapid senescence process characterized by an enrichment in genes mainly involved in oxidative stress and cell wall disassembly, linked to textural and turgor loss after harvest [11,12]. Short-term treatments with exposure to high levels of CO_2_ (15–20 kPa) for 2–3 days at low temperature have been shown to effectively maintain the quality of different berries, increase firmness, and reduce fungal infections during cold storage [13,14,15]. After transfer to 20 °C following cold storage, at the time of consumption, transcriptomic analysis (RNA-seq) of strawberries [12] revealed that pretreatment with high CO_2_ levels in addition to the down-regulation of genes involved in pectin solubilization and depolymerization, there was a strong transcriptional modulation of genes related to cell wall remodeling and glycosylation pathways. In this respect, among the most prominent findings was the significant enrichment of the Gene Ontology term GO:0016772-transferase activity, transferring phosphorus-containing groups, and GO:0016758-hexosyltransferase activity, transferring hexosyl groups.

Since AGPs and extensins are frequent targets of glycosyltransferases, and nucleotide sugars (UDP-sugars) are essential donors in their glycan biosynthesis, this study aimed to perform an integral analysis of UDP-sugar profiling and the transcriptional and post-translational regulation of these structural *O*-glycoproteins in order to better understand their role in cell wall remodeling during softening and their contribution to postharvest storage quality. The specific objectives were as follows: (1) to characterize the accumulation and subcellular localization of extensin and AGP isoforms in Mara des Bois strawberries under different storage conditions; (2) to assess the expression of AGP-encoding genes (*FvAGP4*, *FvAGP5*) and glycosylation-related genes (*FvP4H1*, *FvGAT20*, *FvGAT7*); and (3) to profile the levels of key nucleotide sugars (UDP-galactose, UDP-arabinose, UDP-glucuronic acid, UDP-rhamnose). To achieve this, an effective extraction method was developed, and a sensitive and selective LC-ESI-QTOF-MS/MS protocol was optimized for the accurate quantification of UDP-sugars in fruit tissues.

## 2. Results

### 2.1. Extensins and Arabinogalactan Proteins Pattern During Strawberries Postharvest Storage

**Figure 1:**
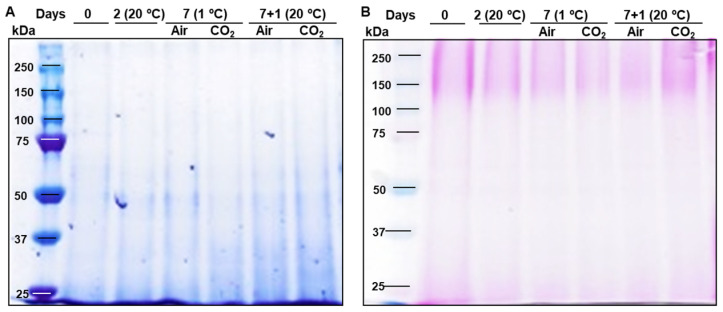
SDS-PAGE (8% polyacrylamide) Coomassie Brilliant Blue staining (**A**) and glycoprotein staining (**B**) of strawberries Mara des Bois proteins (10 µg) extracted from freshly harvested (day 0), stored at 20 °C for 2 days, untreated (Air) and CO_2_-treated (CO_2_) after storage at 1 °C for 7 days and after transfer to 20 °C for 1 day.

Figure 1 shows the results of the electrophoretic analysis and general protein staining using Coomassie Blue, along with specific staining to detect glycoproteins in the extracted protein fractions. The Coomassie-stained gel revealed a higher abundance of polypeptides with molecular mass between 25 and 50 kDa. In contrast, the glycoprotein-stained gel showed more prominent bands in the high-molecular-mass zone. This difference is due to the presence of highly glycosylated proteins, which have a molecular mass above 50 kDa and are less susceptible to Coomassie staining.

**Figure 2:**
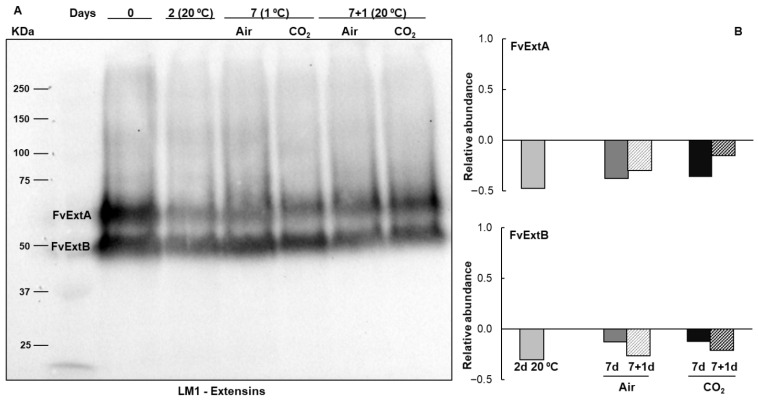
Immunoblot of extensin isoforms in strawberries Mara des Bois freshly harvested (day 0), stored at 20 °C for 2 days, untreated (Air) and CO_2_-treated (CO_2_) after storage at 1 °C for 7 days and after transfer to 20 °C for 1 day. (**A**) Protein extracts (10 µg) were separated by SDS-PAGE (8% polyacrylamide) under reducing conditions, transferred to PVDF membranes, and probed with antisera raised against extensins, LM1. (**B**) Histograms represent the changes in the levels of immunoreactive extensin bands, quantified by scanning densitometry and expressed as the relative fold-change with respect to the levels in freshly harvested fruit (day 0). The results shown are representative of the three biologicals replicated.

**Figure 3:**
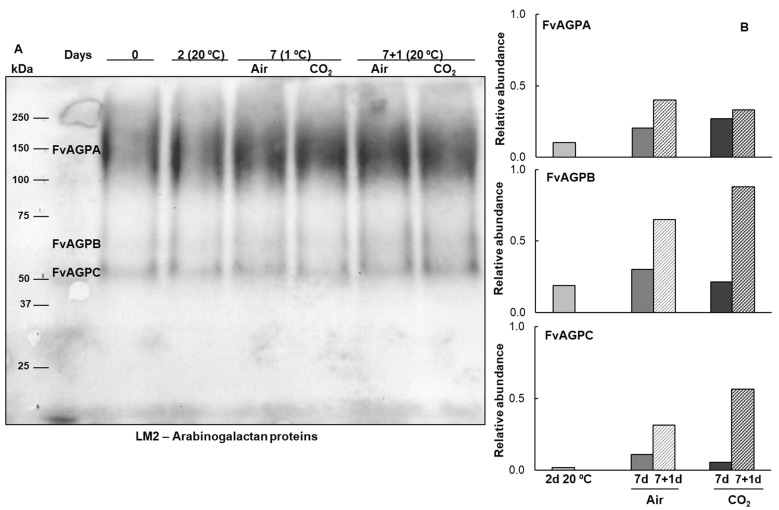
Immunoblot of arabinogalactan proteins (AGPs) isoforms in strawberries Mara des Bois freshly harvested (day 0), stored at 20 °C for 2 days, untreated (Air) and CO_2_-treated (CO_2_) after storage at 1 °C for 7 days and after transfer to 20 °C for 1 day. (**A**) Protein extracts (10 µg) were separated by SDS-PAGE (8% polyacrylamide) under reducing conditions, transferred to PVDF membranes, and probed with antisera raised against AGPs, LM2. (**B**) Histograms represent the changes in the levels of immunoreactive AGP bands, quantified by scanning densitometry and expressed as the relative fold-change with respect to the levels in freshly harvested fruit (day 0). The results shown are representative of the three biologicals replicated.

To determine the presence of *O*-glycosylated proteins belonging to the hydroxyproline-rich glycoprotein (HRGP) family in freshly harvested Mara des Bois strawberries and to study the effect of temperature and different atmospheric storage conditions, extracted proteins were transferred onto PVDF membranes and immunodetected using antibodies against two major *O*-glycosylated protein families: LM1 for extensins and LM2 for arabinogalactan proteins. After harvest, two polypeptides with apparent molecular masses of 67 (FvExtA) and 51 (FvExtB) kDa were detected by the LM1 extensin antisera, and three polypeptides were recognized by the LM2 arabinogalactan protein antisera. One of these was a broad band with an apparent molecular mass ranging from 118 to 192 kDa (FvAGPA). In contrast, the other two are more homogeneous single bands with apparent masses of 70 (FvAGPB) and 58 (FvAGPC) kDa. The expression and accumulation patterns of these proteins are modified by temperature and atmospheric storage conditions (Figure 2 and Figure 3).

The analysis of the immunoblot pattern of extensin isoforms revealed a sharp decrease in their relative abundance during postharvest storage compared to freshly harvested strawberries, especially in non-cold-stored senescent fruit at 20 °C (Figure 2). The decrease in the relative abundance of extensin polypeptides was less pronounced for FvExtB after 7 days of storage at 1 °C and for FvExtA after 1 day at 20 °C, in both untreated and CO_2_-treated fruit (Figure 2).

In contrast, densitometric analysis revealed differential increases in the relative abundance of the three AGP isoforms compared to freshly harvested fruit under all storage conditions. These increases were observed in strawberries stored at 20 °C, as well as in both untreated and treated fruit during postharvest storage and after transfer to 20 °C. Additionally, the three AGP isoforms reached maximum levels when strawberries were transferred to 20 °C following a cold storage period, regardless of whether the fruit had been treated with 18 kPa CO_2_ (Figure 3). This contrasts with the lower relative abundance of the AGP isoforms in non-cold-stored fruit. It is also noteworthy that FvAGPB and FvAGPC showed the greatest increase in relative abundance among the AGPs present in strawberries. A differential effect of high CO_2_ pretreatment was also observed in these isoforms, with treated fruit reaching a higher relative abundance than untreated fruit after transfer to 20 °C. Conversely, the expression level of the FvAGPA isoform was higher in the untreated fruit (Figure 3).

### 2.2. Subcellular Localization of Extensins and Arabinogalactan Proteins in Mara des Bois Strawberries

**Figure 4:**
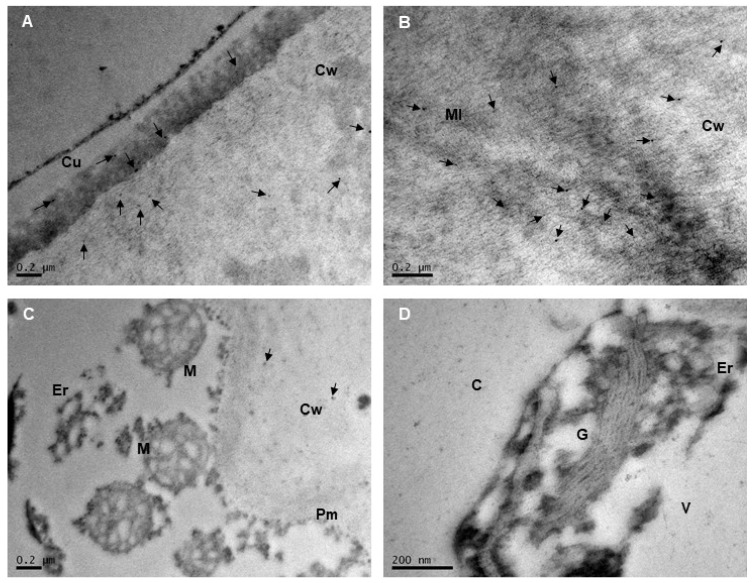
TEM immunolocalization of the extensin epitope recognized by LM1 in pericarp cells of Mara des Bois strawberries. Representative images of subcellular localization from different cell compartments are shown (**A**–**D**). Cell wall (Cw), middle lamella (Ml), cuticle (Cu), plasma membrane (Pm), cytoplasm (C), vacuole (V), mitochondria (M), Golgi apparatus (G), endoplasmic reticulum (Er). Arrows indicate labeling of 10 mm colloidal gold particles. Scale bars represent 0.2 µm. The micrographs shown are representative of six biological samples.

**Figure 5:**
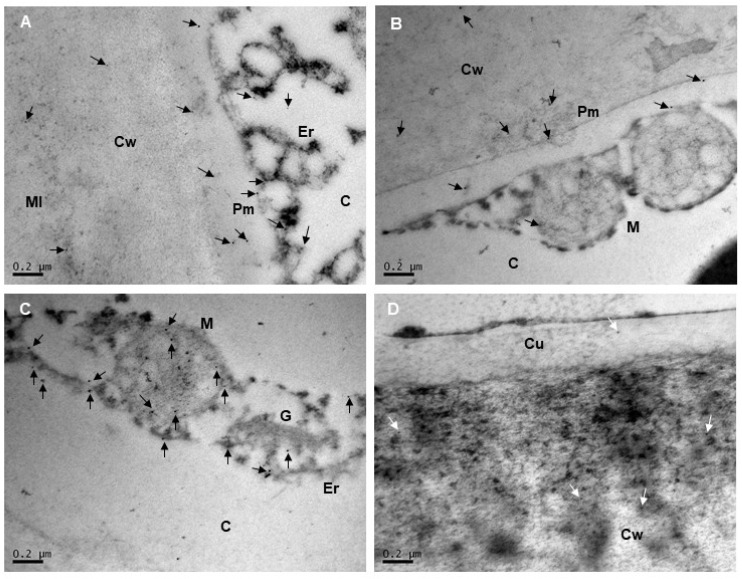
TEM immunolocalization of the AGP epitope recognized by LM2 in pericarp cells of Mara des Bois strawberries. Representative images of subcellular localization from different cell compartments are shown(**A**–**D**). Cell wall (Cw), middle lamella (Ml), cuticle (Cu), plasma membrane (Pm), cytoplasm (C), vacuole (V), mitochondria (M), Golgi apparatus (G), endoplasmic reticulum (Er). Arrows indicate labeling of 10 mm colloidal gold particles. Scale bars represent 0.2 µm. The micrographs shown are representative of six biological samples.

Immunogold labeling was used to visualize the distribution of extensins and AGPs in the cell wall and in various subcellular compartments. The subcellular localization of extensins and AGPs was examined in ultrafine sections of the epidermis and subepidermis of Mara des Bois strawberries using immunogold electron microscopy (Figure 4 and Figure 5). It is important to note that these patterns do not represent the total protein content in the tissue layer, as the protein was heterogeneously distributed within the epidermis and the images were acquired at high magnification. Furthermore, no gold particles were detected when monoclonal antibodies against extensin and AGP epitopes, LM1 and LM2, respectively, were omitted during immunolabeling. This indicated that the localizations identified through immunogold electron microscopy in these experiments were both specific and reliable.

Extensins were predominantly detected throughout the cell wall and, to a lesser extent, in the cuticle of all epidermal cell layers. Within the cell wall and the cuticle, LM1 gold grains were scattered, without forming clusters or associating with osmiophilic elements. In the cell wall, labeling was primarily observed in the region near the cuticle and middle lamella (Figure 4A,B). No labeling was detected at the plasma membrane, vesicles, or on organelle membranes (Figure 4C). Similarly, no hybridization with LM1 antisera was observed in mitochondria, rough endoplasmic reticulum, the Golgi apparatus, or free in the cytoplasm (Figure 4D).

At the subcellular level, AGPs were primarily detected in association with cell membranes, including the plasma membrane and organelle membranes (e.g., those of the mitochondria, rough endoplasmic reticulum, and Golgi apparatus), while no labeling was observed in the cytoplasm (Figure 5A–C). In mitochondria, colloidal gold labeling was present on the outer membrane, and AGPs were also found inside the organelle, in both the matrix and the mitochondrial cristae membranes (Figure 5B,C). On the plasma membrane, AGPs were randomly distributed and did not form clusters at specific locations (Figure 5A,B). AGP epitopes were also detected in the cell wall, with labeling occurring preferentially near the cell wall-plasma membrane continuum and in the cytoplasm (Figure 5A,B), and to a lesser extent in the middle lamella and cuticle (Figure 5D).

### 2.3. Arabinogalactan Protein Genes Expression Pattern During Strawberries Postharvest Storage

In light of our previous RNA-seq study results [12], and to further our understanding of the postharvest regulation of AGPs in Mara des Bois strawberries, we analyzed the transcript levels of two genes that were found to be among the most strongly regulated. *FvAGP4* (*FvH4_1g17080*; XM_004287928.2), which encodes for an arabinogalactan protein 4 with high homology to a subgroup of lysine-rich arabinogalactan proteins in plants, including *AGP9/17/18* from *Arabidopsis*, and *FvAGP5* (*FvH4_1g20060*; XM_011469704.1), which encodes a classical arabinogalactan protein 5-like.

Table 1 presents the amino acid composition and physicochemical properties of the amino acid sequences of proteins encoded by *FvAGP* genes in strawberries. Consistent with classical AGPs [16], both proteins displayed characteristic structural features, including a domain enriched in PAST residues (including sites for Hyp-*O*-glycosylation), which accounted for over 68% of the total amino acid content. Notably, the proteins showed a high abundance of proline and alanine residues, with lysine content exceeding 3% in lysine-rich arabinogalactan protein 4. Additionally, the sequences contained repetitive dipeptides such as Ala-Pro, Ser-Pro, Thr-Pro, and Pro-Ala, as well as an N-terminal hydrophobic signal peptide and a C-terminal hydrophobic sequence directing the addition of a GPI anchor (Table 1). The 815 bp and 926 bp cDNAs for *FvAGP4* and *FvAGP5* encoded deduced sequences of 196 and 133 amino acids, respectively, with isoelectric points of 6.13 and 5.82 and estimated molecular masses of 18.16 and 12.43 kDa. The physicochemical properties of each AGP, as determined by ProtParam, indicated that both proteins were unstable, with instability indices of 83.57 and 77.58, respectively.

However, they were stable over a wide range of temperatures, as evidenced by their high aliphatic indices. The positive GRAVY indices suggest that both proteins are more hydrophobic and less water-soluble. Their secondary structures were composed of 2–3% α-helices, 10–11% folded β-sheets, and 86–88% random coil (Table 1), indicating a high degree of intrinsic disorder for both proteins.

**Figure 6:**
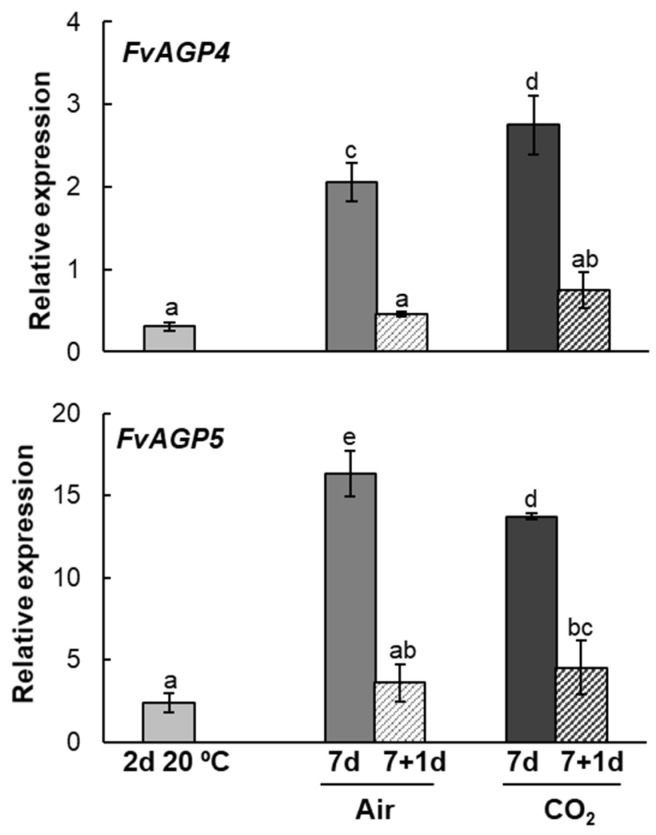
Changes in the expression levels of *FvAGP4* and *FvAGP5* transcripts in strawberries Mara des Bois freshly harvested (day 0), stored at 20 °C for 2 days, untreated (air) and CO_2_-treated (CO_2_) after storage at 1 °C for 7 days and after transfer to 20 °C for 1 day. Transcript levels were estimated by RT-qPCR and normalized using *FvACT* as a reference gene. Results were calculated relative to a calibrator sample (freshly harvested fruit, day 0) using the 2^−ΔΔCt^ method. Data are the mean ± SD, *n* = 6. Different letters indicate significant differences using the Tukey-b test (*p* ≤ 0.05).

Figure 6 shows that the expression patterns of *FvAGP4* and *FvAGP5* were differentially regulated by both temperature and high CO_2_ levels. At the end of 7 days of storage at 1 °C, a higher abundance of both transcripts was observed in untreated and CO_2_-treated strawberries compared to freshly harvested fruit, with CO_2_ having a more pronounced effect on the expression levels of *FvAGP4*. It is noteworthy that *FvAGP4* and *FvAGP5* transcript levels decreased significantly when both untreated and CO_2_-treated strawberries were transferred to 20 °C after cold storage. Low expression levels of both transcripts were also observed in non-cold-stored fruit at 20 °C (Figure 6).

### 2.4. Post-Transcriptional Arabinogalactan Protein Genes Expression Pattern During Strawberries Postharvest Storage

In order to elucidate the relevance of post-translational modifications of AGP glycoproteins during strawberry postharvest storage, we analyzed the expression patterns of key genes involved in these processes. Prolyl 4-hydroxylase 1 (*FvP4H1*; *FvH4_4g11880*; XM_011464281.1), located in the endoplasmic reticulum, catalyzes the conversion of peptidyl-proline to form 4-hydroxy-L-proline. β-1,3-Galactosyltransferase 20 (*FvGAT20*; *FvH4_5g38810*; XM_004293466), which has hydroxyproline *O*-galactosyltransferase activity, transfers galactose from UDP-galactose to the hydroxyproline residues in the arabinogalactan peptide backbone. β-1,3-Galactosyltransferase 7 (*FvGAT7*; *FvH4_3g04990*; XM_011467025.1) catalyzes the transfer of a galactose residue from a donor molecule to an oligosaccharide, forming a β-1,3-linkage. Both galactosyltransferases belong to the glycosyl transferase 31 family and are located in the Golgi membrane.

**Figure 7:**
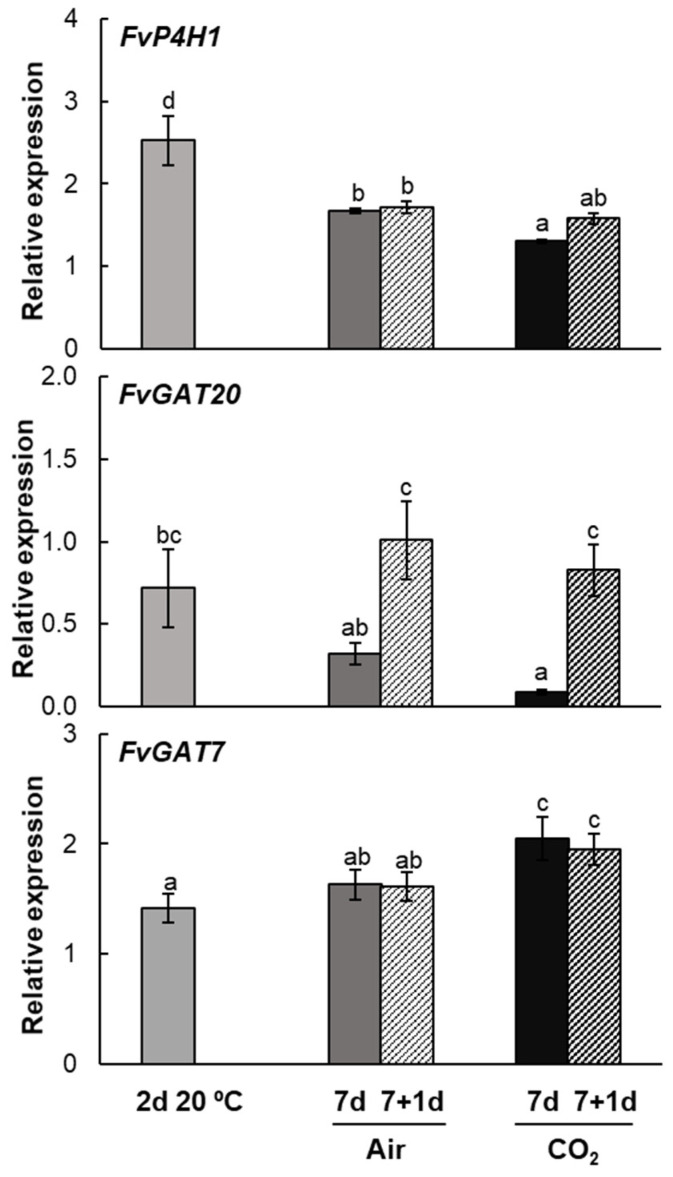
Changes in the expression levels of *FvP4H1*, *FvGAT20* and *FvGAT7* transcripts in strawberries Mara des Bois freshly harvested (day 0), stored at 20 °C for 2 days, untreated (air) and CO_2_-treated (CO_2_) after storage at 1 °C for 7 days and after transfer to 20 °C for 1 day. Transcript levels were estimated by RT-qPCR and normalized using *FvACT* as a reference gene. Results were calculated relative to a calibrator sample (freshly harvested fruit, day 0) using the 2^−ΔΔCt^ method. Data are the mean ± SD, *n* = 6. Different letters indicate significant differences using the Tukey-b test (*p* ≤ 0.05).

Relative gene expression analysis of the *FvP4H1* gene showed an increase in transcript abundance during postharvest storage, with maximum expression observed in non-cold-stored fruit at 20 °C. After 7 days of storage at 1 °C, transcript levels in both untreated and CO_2_-treated fruit were higher compared to freshly harvested fruit, though still lower than in fruit that had not been cold-stored. When strawberries were transferred to 20 °C, *FvP4H1* expression remained similar to levels observed at the end of cold storage in both untreated and CO_2_-treated fruit (Figure 7).

The study of the expression patterns of the β-1,3-galactosyltransferase genes revealed differential regulation by environmental factors such as temperature and high CO_2_ exposure. Regarding the *FvGAT20* gene, transcript levels were down-regulated during cold storage and in non-cold-stored fruit. It is noteworthy that transcript levels increased significantly when fruit stored at low temperatures was transferred to 20 °C, reaching transcript levels comparable to those of freshly harvested fruit (Figure 7). In contrast, *FvGAT7* expression was up-regulated regardless of temperature or postharvest storage conditions, with the highest transcript abundance observed in CO_2_-treated strawberries (Figure 7). When both untreated and CO_2_-treated fruit were transferred to 20 °C, their transcript levels remained similar to those recorded at the end of cold storage.

### 2.5. Identification and Quantification of UDP-Sugars Involved in Arabinogalactan Protein Glycosylation During Strawberries Postharvest Storage

To confirm the identity of the UDP-sugars present in the samples, tandem mass spectrometry (MS/MS) analyses were performed using high-resolution quadrupole time-of-flight (QTOF) mass spectrometry operated in negative electrospray ionization (ESI) mode. Fragmentation was carried out at a fixed collision energy of 20 eV. The samples were previously purified using graphitized non-porous SPE tubes and analyzed by a sensitive and selective LC-ESI-QTOF-MS/MS method specifically developed for the accurate detection of UDP-galactose, UDP-arabinose, UDP-glucuronic acid, and UDP-rhamnose in fruit tissues.

**Figure 8:**
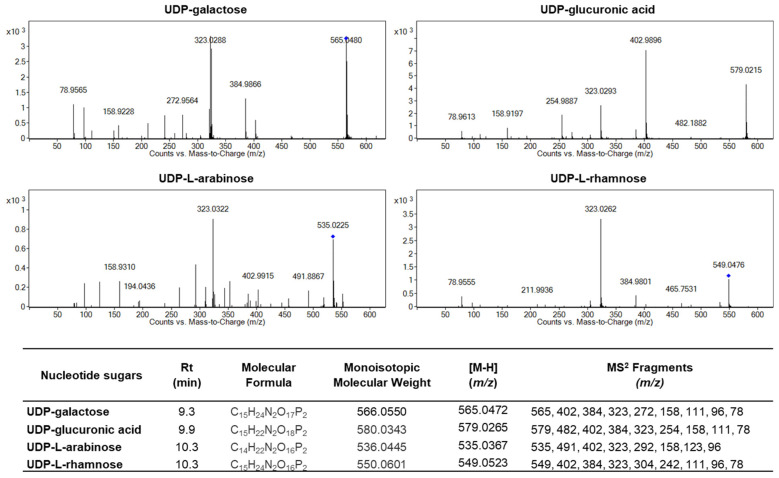
Fragmentation patterns and corresponding MS/MS spectra of the nucleotide sugars in Mara des Bois strawberries.

Figure 8 shows representative MS/MS spectra for the four UDP-sugars. In each case, the observed precursor ion matched the expected [M-H]^−^ mass with high precision: *m*/*z* 565.0480 for UDP-galactose, 579.0215 for UDP-glucuronic acid, 535.0225 for UDP-L-arabinose, and 549.0476 for UDP-L-rhamnose, in close agreement with theoretical values. All four compounds exhibited a major fragment ion at *m*/*z* 323, which corresponds to a conserved UDP-derived fragment, a characteristic and highly conserved fragment among UDP-sugars. Additional fragment ions at *m*/*z* 384, 402, and 158 were also detected and are typical of the UDP backbone. Importantly, each UDP-sugar produced unique diagnostic fragments that allowed their discrimination: UDP-glucuronic acid showed a prominent fragment at *m*/*z* 482, UDP-L-arabinose at *m*/*z* 491, and UDP-L-rhamnose at *m*/*z* 465. Together, the distinct fragmentation patterns, high-resolution mass measurements, and reproducible retention times provided robust analytical evidence for the identification and differentiation of structurally related UDP-sugars in the analyzed fruit samples.

Quantification of endogenous nucleotide sugars revealed that, in freshly harvested strawberries, the most abundant activated sugar was UDP-galactose, present at approximately 0.47 ± 0.03 µg per g of fresh weight. This level was over 1.5 times higher than those of UDP-arabinose and UDP-glucuronic acid and about 1.9 times higher than that of UDP-rhamnose (Table 2). Overall, postharvest storage led to a significant (*p* ≤ 0.05) decrease in nucleotide sugar levels, particularly in non-cold-stored fruit at 20 °C (Table 2). The decline in the UDP-sugars was less pronounced in CO_2_-treated strawberries (Table 2).

## 3. Discussion

The plant cell wall is a dynamic structure involved in key processes throughout the plant life cycle, providing mechanical strength, facilitating the transport of water and nutrients, and supporting cell enlargement and differentiation. It also protects against abiotic and biotic stresses, serving as a barrier to pathogens, and contributes to morphogenesis and plant architecture [17]. These functions are linked to the complex structure of the cell wall, which primarily consists of cellulose, hemicellulose, pectin, and structural proteins [2,18]. Cell wall models highlight the importance of both non-covalent and covalent interactions between structural proteins and polysaccharides in dynamic responses to developmental and environmental changes [19]. Extensins and arabinogalactan proteins (AGPs), two structural *O*-glycosylated hydroxyproline-rich proteins, form complexes with pectin and hemicellulose through ionic interactions and covalent cross-linking [3]. These complexes are essential for cell wall assembly and regulation of mechanical properties, such as turgor pressure, intercellular adhesion, and cell wall integrity [18].

Changes in fruit texture during ripening and senescence are primarily caused by modifications to the cell wall structure and composition. These include depolymerization of the glycan matrix, solubilization and/or depolymerization of pectins, and loss of neutral sugars from pectin side chains. Additionally, the loosening of the xyloglucan-cellulose network and cell wall swelling increases wall porosity, which may facilitate access for degradative enzymes [20,21]. It is well known that short-term treatments with exposure to high levels of CO_2_ at low temperature have been shown to increase strawberries firmness and reduce weight loss in cold storage [13,22,23]. The enhanced resistance to softening induced by short-term high CO_2_ postharvest treatment was associated with the preservation of cell wall elasticity, together with the up-regulation of genes involved in maintaining cell turgor, xyloglucan stabilization, maintenance of expression levels of nucleotide sugar transferases, and improved middle lamella integrity [12]. However, the involvement of structural *O*-glycoproteins in the dynamic cell wall responses associated with strawberry senescence-related processes, as well as in the cell wall architectural modifications induced by short-term high CO_2_ treatment that enhance postharvest storage quality, remains largely unknown.

Although the precise functions of extensins remain unclear, they are known to perform versatile roles in plant development and stress responses. As structural components, these proteoglycans form covalent scaffolds through cross-linking of repetitive tyrosine motifs with pectins, hemicellulose, and other cell wall components, crucial for maintaining cell wall integrity. Additionally, they interact with pectins via ionic bonds in response to mechanical stress [9]. Extensins participate in cytokinesis during early embryo and root nodule development, microspore growth, wound responses, and pathogen resistance [24]. Research on extensins during fruit ripening remains limited. An increase in extensin deposition at the onset of ripening has been associated with an oxidative burst in table grapes, which promotes cross-linking into insoluble forms that enhance resistance to fungal enzymes and modulate pectin structure and primary cell wall hydration [25].

The study of proteins recognized by the monoclonal antibody LM1, raised against extensin glycan epitopes, revealed two constitutive extensin isoforms in Mara des Bois strawberries with apparent molecular masses of 51 and 67 kDa. Immunoblot analysis showed a significant decrease in the relative abundance of both isoforms during postharvest storage, particularly in non-cold-stored senescent strawberries at 20 °C, suggesting a potential association with the progressive loss of cell wall integrity during senescence. Immunogold labeling with LM1 reveals the subcellular localization of extensin epitopes in the cuticle, the subjacent pectic layer (primarily composed of pectic polysaccharides), and the middle lamella of epidermal cell walls. The decrease in extensin levels after harvest in strawberries, crucial for maintaining cell wall structure, could contribute to senescence fruit softening.

AGPs represent a heterogeneous family, with individual members playing diverse roles in plant growth and development. They are involved in processes such as cell proliferation, programmed cell death, cell-to-cell signaling, cell wall plasticity, hormone signaling, plant-microbe interactions, and responses to abiotic stress [3,5,7,26,27]. Studies on AGPs in fruit, both in situ and ex situ, have shown their consistent presence in fruit tissues, where the structural and morphological properties of AGPs, as well as calcium concentrations, correlate with ripening progress and proper fruit cell wall assembly [28]. The first molecular mass reports of AGPs extracted from fruit indicated a range of 55–210 kDa in *Solanum lycopersicum* cv. Ailsa Craig pericarp [29], 70–250 kDa in *Malus domestica* cv. Jonaprince [30], and 20–120 kDa in *Solanum lycopersicum* cv. Betalux [31]. This variation in AGP molecular mass across fruit species and cultivars indicates a diverse AGP pattern. The analysis of proteins recognized by a monoclonal antibody targeting arabinogalactan protein glycan epitopes reveals that Mara des Bois strawberries contain three constitutive AGP isoforms, each exhibiting varying extents of glycosylation. Two isoforms display apparent molecular masses of 58 and 70 kDa, while a third shows a broad band ranging from 118 to 192 kDa. The smeared appearance of these bands reflects the glycomodule heterogeneity characteristic of AGPs. The protein backbones, which account for approximately 10% of the total molecular mass, typically consist of 100–180 amino acid residues. In contrast, the sugar chains, which make up over 90% of the AGP’s molecular mass, range from 30 to 150 sugar residues and retain the arabinogalactan type II (AG II) structure. In this context, variations in glycosylation and UDP-sugar levels influence both the molecular mass and conformation of AGP molecules, which can range from 60 to 300 kDa [16]. Research on AGPs suggests that the protein moiety primarily serves as a scaffold for glycan attachment, with AGP functions being directly dependent on glycosylation. The glycan structures enable interactions with other cell wall glycopolymers, and their attachment influences AGP localization, intracellular distribution, and stability. Extensive glycosylation is known to confer, moreover, resistance to proteolytic degradation, enhance solubility, and improve thermal stability [3,26]. The biosynthesis of these complex glycans requires nucleotide sugars such as UDP-galactose and UDP-arabinose, which serve as activated sugar donors for galactosyl- and arabinosyltransferases, respectively. Since these nucleotide sugars act as direct donors in the glycosylation of AGPs, their intracellular levels are expected to critically influence AGP structure and biological function, which in turn compromises cell wall integrity and plant adaptability [32]. Accurate quantification of UDP-galactose and UDP-arabinose in strawberries under varying physiological and stress conditions is therefore essential for understanding their roles in AGP biosynthesis and potential function. In the present work, we developed a method for extracting nucleotide sugar using cold chloroform-methanol, followed by solid-phase extraction (SPE) for sample cleanup and enrichment, employing cartridges optimized for polar metabolite retention. For chromatographic separation of UDP-sugars, we used electrospray ionization in negative mode (ESI) coupled to a quadrupole time-of-flight mass spectrometer (QTOF-MS/MS) for high-resolution detection. UDP-galactose, UDP-arabinose, UDP-glucuronic acid, and UDP-rhamnose were found in concentrations of 0.2 to 0.5 μg per g of fresh weight in strawberries. Data evaluation revealed a significant decrease (*p* ≤ 0.05) in the four UDP-sugar concentrations in non-cold-stored fruit at 20 °C.

Immunocytochemical analyses have linked glycosylation levels to molecular changes in AGPs during cellular development. Selective glycome profiling revealed a decrease in AGP molecular mass and concentration as ripening progressed. At the breaker stage of tomato fruit, an AGP band of high molecular mass (~60–120 kDa) with various carbohydrate chains is present, whereas low-molecular-mass AGPs (~30 kDa) dominate at the red ripe stage. These smaller AGPs may serve as markers of the end of the ripening process [31]. Similarly, in grape ripening, AGP epitopes initially increase before being released into the apoplast [25]. Immunoblot in Mara des Bois strawberries revealed isoform-specific regulation of AGPs in response to temperature and atmospheric storage conditions. Unlike extensins, non-cold-stored senescent fruit at 20 °C exhibited lower levels of the three AGP isoforms. However, cold-stored fruit transferred at 20 °C showed a significant increase in the low-molecular-mass isoforms, FvAGPB and FvAGPC, which were further up-regulated by CO_2_ pretreatment. This response may be associated with the effect of short-term CO_2_ exposure in delaying senescence-related physiological disorders, thereby enhancing resistance to softening and limiting water loss in strawberries [11,13].

Studies using monoclonal antibodies to immunolocalize AGP epitopes have shown that AGPs are widespread across the plant kingdom, present in cell walls, plasma membranes, apoplastic spaces, secretions, and intracellular multivesicular bodies [3,5,33]. Immunocytochemical analyses further reveal that certain AGP epitopes are associated with endomembranes and secretory compartments, such as the endoplasmic reticulum and Golgi apparatus, in cells actively producing and secreting AGPs [34]. In strawberry epidermal cells, immunogold labeling with LM2 confirmed the localization of AGPs within the cell wall-plasma membrane continuum and endomembranes, particularly the endoplasmic reticulum, Golgi apparatus, and mitochondria. The presence of AGPs in endomembrane compartments emphasizes that the synthesis of carbohydrate chains and the protein moiety takes place in epidermal cells. In the outer tangential epidermal wall, AGPs were found in the innermost region of the non-cutinized cellulose layer, indicating their interaction with cell wall polymers, such as cellulose microfibrils, which are connected by hydrogen bonds to xyloglucans and pectic polysaccharides [35]. Molecular analyses of AGPs in fruit highlight their role in ion binding, maintaining cell wall-plasma membrane integrity, and cross-linking with other cell wall constituents. In this context, classical AGPs are believed to form complexes with pectins and hemicelluloses through covalent bonds with RG-I and HG linked to rhamnose residues in AG polysaccharides, as well as arabinoxylan linked to rhamnose in RG-I [36]. Additionally, calcium-ion-driven interactions facilitate cross-linking between AGPs and acidic pectic polysaccharides, mediated by the carboxyl groups of uronic acid residues in both AGPs and pectins. In this context, the elevated levels of AGP isoforms in CO_2_-treated strawberries, characterized by firmer texture and improved water retention, may reflect the role of CO_2_ in modulating cell wall remodeling, particularly by preserving the cross-linking network involving xyloglucans and pectins, whose destabilization was more effectively controlled by CO_2_ pretreatment [12].

The expression of AGP genes is differentially regulated by developmental factors, growth conditions, and abiotic stresses. The abundance of AGPs, as detected by monoclonal antibodies, and the gene expression profiles of Lys-rich *LeAGP1* and classical AGPs *SlAGP2* and *SlAGP4* were monitored during tomato fruit ripening and in response to mechanical wounding, hypoxia, and anoxia [29]. The polypeptides recognized by these antibodies exhibited constitutive expression during ripening and under hypoxic conditions, with slight up-regulation in response to mechanical wounding and marked up-regulation under anoxia, suggesting their involvement in both ripening and stress responses. In banana, classical *MaAGP1/2/6/9/16/17* genes were differentially up-regulated in response to low-temperature stress, particularly in the stress-tolerant cultivar [37]. Similarly, AGP epitopes have been shown to increase in apples, potentially forming a mechanical barrier that protects against pathogen invasion [38]. Comparative transcriptome and weighted gene co-expression network analysis of Mara des Bois strawberries under different storage conditions revealed that AGP genes and nucleotide sugar transferases were included in the huge genes associated with cell wall structural architecture in firmer CO_2_-treated fruit [12]. In this work, we established the expression patterns of two RNA-seq-identified arabinogalactan protein genes, *FvAGP4*, which shares high homology with plant Lys-rich AGPs, including *AGP9/17/18* from *Arabidopsis*, and *FvAGP5*, which encodes a classical AGP5-like protein. Both genes were significantly up-regulated by low temperature and low temperature-high CO_2_ levels. The amino acid composition and physicochemical properties of the proteins encoded by the *FvAGP* genes classify them as classical AGPs [26,39]. Both proteins feature a high proportion of proline, alanine, serine, and threonine (PAST) residues, with Ala-Hyp, Ser-Hyp, and Thr-Hyp dipeptide repeats. *FvAGP4* also has a higher lysine content compared to *FvAGP5*. Both proteins exhibit an N-terminal hydrophobic secretion signal sequence and a C-terminal GPI lipid anchor. Additionally, these proteins show high thermal stability and structural disorder. These unique structural features enable AGPs to be secreted into the extracellular matrix via the endoplasmic reticulum and Golgi apparatus, then delivered to the cell surface, where they attach to the outer leaflet of the plasma membrane facing the cell wall. They also interact with other cell wall and plasma membrane components, regulating the integrity of the cell wall-plasma membrane continuum [40]. Alternatively, AGPs can be cleaved by phospholipases, releasing them from the plasma membrane into the cell wall and extracellular matrix [41], positioning them as key candidates for signal perception and transduction [27]. In addition to acting as structural proteins that mediate cross-linking between cell wall polysaccharides and integrate the plasma membrane with the cell wall, GPI-anchored AGPs may function by accumulating calcium ions in the cell wall matrix via their β-linked glucuronic acid residues in a pH-dependent manner. These calcium ions are then released into the matrix in low pH environments, where they regulate pectin cross-linking and facilitate calcium-dependent signaling pathways in the cell wall [42].

Another interesting aspect is their potential involvement in plant growth regulator signaling, which could influence ripening and senescence. In this sense, overexpression of the tomato *LeAGP1* gene reduces apical dominance, suppresses flower maturation and fruit production, and delays senescence, suggesting an altered balance between auxin and cytokinin signaling [27]. Hormonal regulation of AGP gene expression in other tissues indicates that AGPs may act antagonistically to ABA, modulating cell surface receptors or binding growth factors like ABA [43]. Given that hormones such as ABA and auxins, and specifically the ABA/auxin ratio, could coordinate softening events in strawberry fruit, understanding the relationship between AGPs and hormonal signaling could offer insights into how cell-surface proteins modulate processes regulated by plant growth factors [21].

Our results show a marked difference between the immunoblot pattern of glycosylated AGPs and the expression pattern of *FvAGP* genes in Mara des Bois strawberries. This discrepancy suggests transcriptional regulation of AGP cores by low temperature and low temperature-high CO_2_, which does not correlate with an increase in proteoglycan levels in fruit transferred to 20 °C. Consequently, we are investigating the regulation of key genes involved in the post-translational modification of this *O*-glycosylated protein family in strawberries. In this context, the expression pattern of *FvP4H1*, encoding prolyl 4-hydroxylase that converts proline into hydroxyproline, revealed active regulation of this hydrolase during postharvest storage, particularly in non-cold-stored senescent fruit. The suppression of prolyl 4-hydroxylase activity due to the silencing of *P4H* genes leads to a reduced or complete absence of AGPs. Without proline hydroxylation, AGPs fail to undergo glycosylation, resulting in altered synthesis and subsequent degradation or conversion into lower molecular mass polypeptides [6,29]. The step following proline hydroxylation in AGP biosynthesis is conducted by Hyp-*O*-galactosyltransferase, which adds the first UDP-galactose molecule to the Hyp residue, initiating the subsequent addition of further glycan units to the AGP backbone. In Mara des Bois strawberries, the expression of *FvGAT20*, encoding β-1,3-galactosyltransferase 20, which exhibits hydroxyproline *O*-galactosyltransferase activity, was up-regulated in fruit stored at 20 °C after cold storage. This suggests the reactivation of the initial step in AG glycomodule biosynthesis when strawberries were transferred to ambient temperature. However, the constitutive expression of *FvGAT7*, β-1,3-galactosyltransferase 7, which catalyzes the transfer of a UDP-galactose forming a β-1,3-linkage, supports the notion that the initial transfer of UDP-galactose by β-1,3-galactosyltransferase 20 is a critical step in the post-translational regulation of AGPs in strawberries during postharvest storage. Biochemical analysis of knockout mutants for *Hyp-O-GALT* genes in *Arabidopsis* revealed significant reductions in AGP-specific Hyp-*O*-GALT activity. Further phenotypic analysis of these mutants showed reduced root hair growth, impaired seed coat mucilage production, decreased seed set, and accelerated leaf senescence, highlighting the essential role of AG polysaccharides in AGP function [44].

Galactose residues in AGPs play structural roles beyond glycoprotein architecture. In *Arabidopsis*, reduced galactose in type II AG chains alters seed coat mucilage by disrupting polysaccharide interactions and pectin methylesterification [45]. Assuming UDP-galactose levels reflect AGP glycosylation, its marked decrease in non-cold senescent strawberries kept at 20 °C may lead to structural changes and impaired AGP function in senescence signaling. Additionally, part of the UDP-galactose reduction may result from its conversion to UDP-glucose, highlighting the metabolic interplay between nucleotide sugars under stress [46]. UDP-galactose also contributes to raffinose-family oligosaccharide biosynthesis via galactinol, and its levels, along with galactinol and raffinose, are affected by stress [11,13]. These shifts suggest that altered UDP-sugar availability may influence multiple pathways relevant to fruit quality. Monitoring UDP-sugar dynamics could thus provide biochemical markers of stress tolerance and offer insights into respiratory metabolism, with implications for postharvest storage quality and breeding strategies.

## 4. Materials and Methods

### 4.1. Plant Material and Postharvest Treatments

*Fragaria* sp. strawberries (cv. Mara des Bois) were harvested from a commercial crop in San Sebastián de los Reyes (Madrid, Spain). Ripe strawberries immediately after harvest (0 days) were separated into 25 boxes (5 for each postharvest storage condition) of 200 g of fruit and stored in the dark in chambers with a relative humidity of 80%, under the following conditions: 2 days in air at 20 °C (20 °C), 7 days in air at 1 °C (air), 2 days in a gas mixture containing 18 kPa CO_2_ + 18 kPa O_2_ + 64 kPa N_2_ and 5 days in air at 1 °C (CO_2_), 7 days in air at 1 °C + 1 day at 20 °C (air-transferred), and 2 days at 18 kPa CO_2_-5 days in air at 1 °C + 1 day at 20 °C (CO_2_-transferred). After postharvest treatments, boxes (90 strawberries) from freshly harvested fruit and each storage condition were divided into 3 batches of 30 berries and frozen in liquid nitrogen and stored at −80 °C until analysis.

### 4.2. Isolation of Protein Fraction and Immunoblotting

Total protein fraction was extracted three times from each sample under denaturing conditions. Frozen and liquid nitrogen ground tissue (10 g) was homogenized for 2 min with a Waring blender in 30 mL of 62.5 mM Tris-HCl extraction buffer pH 6.8 containing 20 mM EDTA, 2% CaCl_2_, 1% Triton X-100, 20 mM sodium ascorbate, 2% SDS, 5% β-mercaptoetanol, commercial protease inhibitor cocktail (Roche), and 3% (*w*/*v*) PVPP. After stirring for 30 min, the homogenate was boiled for 10 min and subsequently centrifuged at 12,000× *g* for 40 min at 4 °C, and the resulting supernatant was desalted and concentrated by ultrafiltration through an Amicon Ultra centrifugal 30 K filter device (Millipore). The material retained was precipitated with ice-cold 100% ethanol 1:9 (*v*/*v*) overnight at −20 °C and centrifuged at 12,000× *g* for 30 min. The resulting pellet was dried under nitrogen and resuspended in 62 mM Tris-HCl or Laemmli sample buffer in reducing conditions, containing 5% β-mercaptoethanol, before being analyzed by SDS-PAGE and Western blot. Protein concentration was quantified by the Bicinchoninic Acid method (BCA, Protein Assay Kit, Novagen), a highly sensitive colorimetric assay that is compatible with detergent-solubilized protein solutions, using bovine serum albumin as a standard.

Proteins were separated by SDS-PAGE (8% polyacrylamide) under reducing conditions. Triplicate gels were prepared, one to transfer to PVDF membranes (Amersham) and the others for staining with Coomassie Brilliant Blue or Glycoprotein Staining Kit (Pierce™). The molecular mass of the separated polypeptides was estimated in comparison to the mobility of pre-stained 5 to 250 kDa molecular mass range standard proteins (Precision Plus Protein, Bio-Rad). The membranes were probed with LM1 diluted 1:200 (*v*/*v*) and LM2 diluted 1:100 (*v*/*v*) rat monoclonal antibodies raised against extensin glycan and β-linked-GlcA in AGP glycan epitopes, respectively (Plant Probes), and detected with a goat anti-rat IgG horseradish peroxidase conjugated antibody diluted 1:6000 (*v*/*v*) (Pierce™). Immuno-complexes were visualized using a chemiluminescence (ECL^®^) detection system (Pierce™) and ChemiDoc™ XRS+ gel analysis and documentation equipment with ImageLab™ Software (Bio-Rad, version 6.1), and quantified by scanning densitometry. The results were expressed as the relative fold-change with respect to the levels in freshly harvested fruit (day 0). Independent experiments were performed in at least triplicate.

### 4.3. Immunogold Electron Microscopy

The subcellular distribution of arabinogalactan proteins in the epidermal and subepidermal strawberry cells by immunogold labeling transmission electron microscopy (TEM) was performed as described previously by Vazquez-Hernandez et al. [47]. Briefly, six freshly harvested fruits were cut transversely into 1 mm thick disks, and rectangular pieces (3 mm width × 5 mm length) were immediately fixed for 2 h at 4 °C in PBS pH 7.2 with 1% (*v*/*v*) glutaraldehyde and 4% (*v*/*v*) paraformaldehyde. The tissues were dehydrated in a graded ethanol series, 30–100% (*v*/*v*) for 30 min each time, embedded in LR-White resin (London Resin Company, London, UK) overnight, and polymerized at 60 °C for 48 h. Ultrathin sections (60–70 nm) were cut with a diamond knife on an ULTRACUT E ultramicrotome (Reichert-Jung, Austria) and collected on 100-mesh Formvar-coated nickel grids. Grids containing ultrathin sections were sequentially floated in 0.1 M glycine and 5% BSA. Grids were incubated overnight at 4 °C with LM2 rat monoclonal antibody raised against AGP glycan epitopes diluted 1:10, washed, and incubated for 1 h at room temperature with the secondary antibody, goat anti-rat IgG conjugated to 10 nm gold particles (Sigma-Aldrich, St. Louis, MO, USA) diluted 1:50. Sections were stained with 2% aqueous uranyl acetate and Reynolds’ lead citrate and examined using a JEOL JEM 1010 Transmission Electron Microscope (Tokyo, Japan) at an acceleration voltage of 100 kV. Controls were included by excluding the primary antibody. Four sections were cut from each biological sample and several cells in each section were analyzed (ICTS National Center of Electron Microscopy facility, Complutense University, Madrid, Spain). The micrographs shown are representative of the results obtained from six biological samples.

### 4.4. Relative Gene Expression by Semi-Quantitative and Quantitative RT-PCR

Total RNA of three biological replicate samples was extracted according to Yu et al. [48] with some modifications. Briefly, frozen and liquid nitrogen ground tissue (0.3 g) was transferred to a 1.5 mL centrifuge tube containing 1 mL pre-warmed (65 °C) extraction buffer (2% CTAB, 2% PVP-40, 300 mM Tris-HCl pH 8.0, 2 M NaCl, 25mM EDTA pH 8.0, 0.005% spermidine trihydrochloride, and 2% β-mercaptoethanol), incubated at 65° C for 10 min and centrifuged at 12,000× *g* for 5 min. An equal volume of chloroform/isoamyl alcohol (24:1) was added to the supernatant, centrifuged, and RNA from the upper phase was precipitated with 0.5 volumes of 8 M LiCl overnight at 4 °C, and the pellet was washed with 75% ethanol, air-dried, and re-suspended in 50 μL DEPC-treated water. Total RNA was treated with DNase I recombinant-RNase free (Thermo Fisher Scientific, Waltham, MA, USA) for genomic DNA removal.

The cDNA was prepared by reverse transcription of 1 μg of total RNA using the Maxima cDNA Kit with a dsDNase kit (Thermo Fisher Scientific) following the manufacturer’s instructions. Quantification was performed by real-time quantitative RT-PCR (qPCR) using iCycler iQ^™^ Real-Time PCR Detection System (Bio-Rad) and quantified using the Real-Time Detection System Software (version 2.0). The amplification reactions were carried out in a final volume of 24 μL containing 12 μL of NZY qPCR Green Master Mix (2×) (NZYTech, Ltd.), 2 μL of each primer (10 μM), 2 μL of the cDNA, and 8 µL of water. The PCR profile used was 2 min at 50 °C, 95 °C for 10 min, followed by 40 amplification cycles of 5 s at 95 °C and 20 s at 55–58 °C. Three technical replicates were made from each of the genes studied. Gene expression was determined by the 2^−ΔΔCT^ method using the *F. vesca* Actin-97-like (*FvH4_7g22410*, XM_004307470) as a housekeeping gene [12]. The specific primers used are described in Supplementary Table S1, and PCR amplicons were sequenced to confirm specificity. The results were expressed as the relative fold difference from the expression present in freshly harvested fruit (day 0).

### 4.5. Extraction and Analysis of UDP-Sugars by MS and MS^2^

An effective extraction method and a sensitive and selective LC-ESI-QTOF-MS/MS protocol for the accurate quantification of UDP-galactose, UDP-arabinose, UDP-glucuronic acid, and UDP-rhamnose in fruit tissues have been developed. Nucleotide sugars were extracted following the method of Arrivault et al. [49]. Briefly, frozen, ground plant tissue (50–100 mg) was mixed with ice-cold chloroform-methanol (3:7, *v*/*v*) and incubated at −20 °C for 2 h. Water-soluble compounds were extracted with cold water, followed by centrifugation at 20,000× *g* for 5 min at 4 °C. The aqueous-methanol phase was collected and combined with a second aqueous extract from the chloroform phase. The pooled extracts were diluted with cold water, frozen in liquid nitrogen, and freeze-dried.

Samples were purified using Supelclean^TM^ ENVI-Carb^TM^ SPE Tubes (3 mL, 250 mg, pk 54) (Sigma-Aldrich), following the protocol described by Behmüller et al. [50] with modifications. Briefly, columns were conditioned with 80% acetonitrile in 0.1% trifluoroacetic acid and ultrapure water. Samples, diluted in 10 mM ammonium bicarbonate, were loaded onto the columns at 4 °C. After sequential washes with water, 25% acetonitrile, and 50 mM triethylammonium acetate (TEAA) buffer (pH 7.0), nucleotide sugars were eluted with 25% acetonitrile containing 50 mM TEAA. Resprep^®^ Vacuum Manifolds for SPE sample preparation, filtration, and elution are used. The eluates were supplemented with water, frozen in liquid nitrogen, and freeze-dried. Final samples were reconstituted in ice-cold water, filtered, and stored at −80 °C for HPLC analysis.

The identification of UDP-sugars was made by quadrupole time-of-flight (QTOF) mass spectrometry (QTOF LC-MS/MS apparatus with ESI-Jet Stream Technology, Agilent Technologies) as described by Blanch et al. [13] with modifications. A 10 μL sample was separated on a Tracer Excel C8 120 ODS-B column (25 cm × 0.46 cm, 5 µm particle size, Teknokroma) and eluted with a gradient mobile phase made up of 0.1% formic acid in water (solvent A) and 0.1% formic acid in acetonitrile (solvent B) at a flow of 0.5 mL min^−1^. The solvent gradient changed as follows: 0 min 100% A; 0–30 min 50% A and 50% B; 30–32 min 0% A and 100% B; 32–35 min 100% A. Q-TOF acquisition achieved a high resolution (2 GHz) in MS1 acquisition mode (Min Range *m*/*z* 100, Max Range *m*/*z* 1000). Ionization was achieved by atmospheric pressure electrospray ionization (ESI) with a drying gas flow rate of 12 L min^−1^ at 350 °C, a sheath gas flow of 6.5 L min^−1^ at 300 °C, a nebulizer at 45 psi, a cap voltage of 4000 V, a nozzle voltage of 0 V, a fragmentor voltage of 200 V, and a skimmer 1 voltage of 65 V. The experiments were carried out at negative polarity with a constant collision energy of 20 eV for auto MS^2^ experiments, using the Data Acquisition (version B.04.01) and Qualitative Analysis (version B.04.00) of the MassHunter Workstation software (Agilent Technologies, version B04.01 and B.04.00). The peaks with *m*/*z* 565, 579, 535, and 549 were selected in the ion chromatogram that correspond to UDP-galactose, UDP-glucuronic acid, UDP-arabinose, and UDP-rhamnose, respectively. UDP-galactose was identified with authentic standards, while the other three UDP-sugars were identified by their exact mass, retention time, and MS^2^ fragmentation pattern in negative mode. These compounds were quantified from the areas of their chromatographic peaks in EIC mode by comparison with the calibration curves obtained with the UDP-galactose external standards purchased from Sigma. The compounds analyzed were expressed in µg per g fresh weight, and the data represent the means of the three biological replicates.

### 4.6. Bioinformatic Analysis

Expasy’s Protparam server (http://web.expasy.org/protparam/, accessed on 3 September 2025) was used for the detection of the physicochemical properties of these proteins. The number of amino acids, molecular mass, theoretical isoelectric point (p*I*), instability index, aliphatic index, and grand average of hydropathy index (GRAVY) were calculated for each protein on the basis of amino acid sequences. Protein secondary structure was predicted using the Garnier–Osguthorpe–Robson (GOR) (https://npsa-prabi.ibcp.fr/cgi-bin/npsa_automat.pl?page=npsa_gor4.html, accessed on 3 September 2025) online service. Estimation of intrinsic protein disorder was performed online using the IUPred (http://iupred.enzim.hu/, accessed on 3 September 2025) web server. The proteins encoded by the *FvAGP4* and *FvAGP5* genes were subjected to amino acid analysis, and their polypeptide length, proline, alanine, serine, and threonine (PAST) percentage, and number of AP, PA, SP, and TP repeats were determined. Proteins were identified using the InterProScan software (version 5.75-106.0, https://www.ebi.ac.uk/interpro/search/sequence/, accessed on 3 September 2025). Further, they were subjected to analysis for the presence of signal peptide cleavage sites at the N-terminus and GPI anchor modification sites at the C-terminus using the SignalP 5.0 (https://services.healthtech.dtu.dk/services/SignalP-5.0/, accessed on 3 September 2025) and big-PI Plant Predictor (https://mendel.imp.ac.at/gpi/plant_server.html, accessed on 3 September 2025) tools, respectively.

### 4.7. Statistical Analysis

One-way ANOVA was performed using SPSS Statistics ver. 29.0. (IBM Corporation). Multiple comparisons of means were performed using the Tukey-b test, with a significance level of *p* ≤ 0.05.

## 5. Conclusions

This study establishes that the *O*-glycoproteins, extensins, and arabinogalactan proteins are constitutively expressed in Mara des Bois strawberry tissues and are differentially regulated by temperature and atmospheric conditions during postharvest storage. These regulatory differences likely reflect their specific structural and molecular features, which are potentially linked to their specific roles in cell wall architecture and remodeling. Extensin isoforms are down-regulated after harvest, a trend likely associated with the progressive loss of cell wall integrity and firmness during senescence. In contrast, AGP isoforms remain active throughout postharvest storage and are subject to dual levels of regulation: transcriptionally, in response to low temperature and low temperature-high CO_2_, and post-translationally after transfer to 20 °C, when metabolism resumes after cold storage. UDP-galactose levels and their transfer to hydroxyproline residue by β-1,3-galactosyltransferase 20 emerge as key factors in the post-translational regulation of AGPs. These molecules support AGP glycosylation and, by extension, their structural stability and functional performance. Based on their subcellular localization, molecular properties, isoform glycosylation patterns, UDP-sugar availability, and timing-regulated expression, AGPs appear to play a critical role in maintaining cell wall assembly and modulation of mechanical properties during storage. AGPs may contribute to the preservation of fruit texture and the mitigation of senescence-associated disorders, enhancing resistance to softening and limiting water loss, potentially through CO_2_-responsive signaling pathways. The marked decline in UDP-galactose levels observed in non-cold-stored senescent strawberries correlates with reduced availability of sugar donors for AGP glycosylation, suggesting an AGP structural alteration and impaired function. Further research is needed to better characterize the functional diversity of AGP isoforms, particularly the impact of glycosylation on their subcellular localization, structural behavior, and signaling capacity within the cell wall matrix and cell wall-plasma membrane continuum. In parallel, assessing the dynamics of UDP-sugar levels may help establish their potential as reliable metabolic markers of the respiratory status of fruit under different stress conditions.

## Figures and Tables

**Table 1:** Amino acid composition and physicochemical properties of amino acid sequences encoded by *FvAGP* genes in strawberries.

	*FvAGP4*	*FvAGP5*
total aa/calculated mol mass (kDa)/p*I*	196/18.16/6.13	133/12.43/5.82
proline (P)	47 (24.0) ^a^	25 (18.8)
alanine (A)	49 (25.0)	32 (24.1)
serine (S)	19 (9.7)	15 (11.3)
threonine (T)	19 (9.7)	19 (14.3)
lysine (K)	6 (3.1)	1 (0.8)
PAST (%)	68.4	68.5
AP dipeptide repeats	16	9
PA dipeptide repeats	15	11
SP dipeptide repeats	7	6
TP dipeptide repeats	9	5
N-terminal Signal peptide	Y	Y
C-terminal GPI	Y	Y
aliphatic index (thermostability)	71.58	72.26
instability index	83.57	77.58
grand average of hydropathicity (GRAVY)	0.253	0.431
alpha helix (%)	2.55	3.01
beta sheet	10.20	10.53
random coil (%)	87.24	86.47
Subcellular location	Extracellular—Cell membrane	Extracellular—Cell membrane

^a^ Number in parenthesis are expressed on a percentage basis.

**Table 2:** Nucleotide sugar concentration, expressed as µg g^−1^ fresh weight, in Mara des Bois strawberries after harvest and under different postharvest storage conditions.

	0 Days (20 °C)	2 Days (20 °C)	7 Days (1 °C) + 1 Day (20 °C)	
			Air	CO_2_
UDP-galactose	0.47 ± 0.03 c	0.22 ± 0.02 a	0.37 ± 0.02 b	0.38 ± 0.04 b
UDP-L-arabinose	0.30 ± 0.01 c	0.22 ± 0.01 a	0.24 ± 0.01 ab	0.25 ± 0.01 b
UDP-glucuronic acid	0.30 ± 0.01 c	0.21 ± 0.00 a	0.26 ± 0.01 b	0.26 ± 0.01 b
UDP-L-rhamnose	0.25 ± 0.01 c	0.21 ± 0.00 a	0.23 ± 0.00 b	0.24 ± 0.01 bc

Data represent the mean ± standard deviation and each letter within rows indicates the significant differences between means determined with a Tukey-b test (*p* ≤ 0.05).

## Data Availability

Data are contained within the article or Supplementary Materials.

## Supplementary Materials

The following supporting information can be downloaded at https://www.mdpi.com/article/10.3390/plants14172796/s1: Supplementary Table S1: List of primer sequences used in this study, including the efficiency and the amplicon size.
