# Supplementary material for: A Regulatory Network of Arabinogalactan Proteins, Glycosylation, and Nucleotide Sugars for Optimizing Mara des Bois Strawberries Postharvest Storage Quality
Source: Plants (Basel). 2025 Sep 6;14(17):2796. doi: 10.3390/plants14172796 (PMC12430158; doi:10.3390/plants14172796)
Supplement: Supplementary file 1 [file plants-14-02796-s001.zip › plants-3807930-supplementary.pdf]

Table S1. List of primer sequences used in this study including the efficiency and the amplicon size.

NAME	SEQUENCE	AMPLICON SIZE (bp)	GENE
FvH4_1g17080_FvAGP4_F<br>FvH4_1g17080_FvAGP4_R	CCCGCTATTCTCGGACCAC<br>AAACCCCATCCCAACGCAAT	152	*Arabinogalactan protein 4-like*<br>*FvH4_1g17080*
FvH4_1g20060_FvAGP5_F<br>FvH4_1g20060_FvAGP5_R	TATCTCCTCCACACCGACCT<br>GAAGAAGCTCCCCACCAAAG	145	*Arabinogalactan protein 5-like*<br>*FvH4_1g20060*
FvH4_5g38810_FvGAT7_F<br>FvH4_5g38810_FvGAT7_R	AAGTTTGGTGAGGCGGGAAA<br>CCTTCCACTCGCAATCTGGT	220	*β-1,3-Galactosyltransferase 7*<br>*FvH4_5g38810*
FvH4_3g04990_FvGAT20_F<br>FvH4_3g04990_FvGAT20_R	CGCATGGCAGTGAGGAAAAC<br>ATGCTGCCGTCACATTCTGA	232	*β-1,3-Galactosyltransferase 20*<br>*FvH4_3g04990*
FvH4_4g11880_FvP4H1_F<br>FvH4_4g11880_FvP4H1_R	CTTGAAACGAGGTGGTCAGC<br>CATCCAGACCCATGCTCCAG	188	*Prolyl 4-hydroxylase 1*<br>*FvH4_4g11880*
FvACT_F<br>FvACT_R	ACGAGCTGTTTTCCCTAGCA<br>CTTTTGGATTGAGCCTCGTC	105	*Actin-97-like*<br>*FvH4_7g22410*
